# A Comparative Study of Online Depression Communities in China

**DOI:** 10.3390/ijerph17145023

**Published:** 2020-07-13

**Authors:** Jingyun Tang, Guang Yu, Xiaoxu Yao

**Affiliations:** School of Management, Harbin Institute of Technology, Harbin 150001, China; tang_jing_yun@126.com (J.T.); xiaoxu.yao06@gmail.com (X.Y.)

**Keywords:** depression, online depression communities, negative emotion, social support, participation patterns

## Abstract

Online communities have become a tool for researchers to understand and help individuals with depression. According to their operation mode in terms of management, communities can be divided into management depression communities (MDCs) and lacking-management depression communities (LDCs). This study aimed to investigate the characteristics and impact of LDCs in comparison with MDCs. All postings from the previous year were collected from the LDC and MDC. Keywords were extracted and coded to identify the themes, and a text classifier was built to identify the type of emotions and social support expressed in the postings. Community members were then clustered to explore their different participation patterns. We found that in the LDC, the expression of negative emotions was the most popular theme, there was a lack of information about the treatment of depression and a lack of social support providers, the level of engagement of providers was low, and support seekers did not receive attention. These results reveal the need for community management and can be used to develop more effective measures to support members of online depression communities.

## 1. Introduction

Online communities provide convenient platforms for individuals to communicate with each other. Users gather in online communities because of common interests, goals, or needs [1,2], and can also include members with the same mental illness, such as depression.

Depression is a mental illness affecting more than 350 million people in the world [3]. According to a report issued by the WHO [3], depression is the leading cause of disability worldwide. However, only 25% of the global population with depression receive effective treatment [4]. Stigma is one of the main reasons that prevents patients from receiving treatment [5]. The anonymity and openness of online communities can make people with depression more willing to seek information about mental health problems from those with the same disorder, as they may feel it is less risky to disclose their disorder to others online [5,6]. Online communities provide a platform for people with depression not only to communicate with others and seek information and suggestions, but also to obtain and provide support.

Existing research has explored the feasibility of using online communities to understand and help people with depression. These studies mainly focused on the characteristics [7,8] and participation patterns of members [7,9], the content [10] and nature of postings [11,12], and the impact of participation [13,14]. For example, an analysis of posting content was conducted to investigate themes discussed by members [10], and participation patterns were evaluated to understand how members used the online community. Most of the studies on participation patterns were based on community members as a homogeneous group. For instance, the time spent by members in online depression communities and their posting frequency were investigated [9]. In addition, the motivation and influencing factors of members’ participation in these communities were also explored. [15,16]. On the other hand, some studies examined the differences between member subgroups within communities. For example, research has compared heavy users with less frequent users of communities [17], established the associations between demographic characteristics of members and community activity [7], and examined the participation patterns of different groups of members according to psychological measures [18].

Negative emotions are one of the most significant characteristics of people with depression [11,19]. Previous studies used some methods, such as calculating the proportion of emotional words used [20,21,22], or established text classification model [23,24,25] to classify posts to measure users’ emotions. They found that frequent expression of negative emotions in online communities is associated with higher levels of depression symptoms [26,27,28]. Furthermore, the feasibility of using negative emotions expressed in communities to predict depression was explored [29]. Although a knowledge of the difference in users’ emotions is essential for understanding their behavior in online depression communities, the participation patterns of users with different emotions still need to be investigated.

Seeking social support is considered the main purpose of members’ participation in online health communities [30]. Shumaker and Brownell define social support as “an exchange of resources between at least two individuals perceived by the provider or the recipient to be intended to enhance the well-being of the recipient” [31]. Social support is widely considered to be a protection against depression; consequently, a lack of social support presents a greater risk of depression [32]. Studies on the nature of posts have suggested that the members of online depression communities provide and receive various kinds of social support—most commonly emotional support, followed by informational support [9,33]. Emotional support refers to acknowledging or validating others’ feelings or providing comfort and encouragement [16,34]. Such support can help members reduce the levels of stress or anxiety [33]. Research found that receiving emotional support can also increase members’ community use [16]. In such online communities, informational support has been shown to help members build their network and gain health-relevant knowledge [35]. Such support can promote members’ active response to depression, which may improve the depression symptoms [36]. Social support has an interpersonal approach—that is, individuals can both give and receive social support. However, the relationship between the role of members in social support and their participation patterns in online depression communities has not yet been explored.

Online communities can provide timely social support and overcome geographical restraints [30]. The anonymous nature of online communities makes participants feel secure [5,6]. Therefore, research has generally indicated that online communities have a positive effect on people with depression [13,37,38,39]. However, most of these results are based on management depression communities (MDCs), which feature community managers and support groups, and most are only open to English speakers. Because of China’s unique cultural background, individuals are more ashamed than Westerners in disclosing their depression [40]. The scarcity of mental health services and resources in China leads to 90% of individuals with depression not being treated [41]. Therefore, a large number of people with depression gather on Chinese online communities. However, China’s online depression communities are still in their infancy and have numerous management problems. For example, some Chinese online depression communities do not have managers; therefore, we named them “lacking-management depression communities” (LDCs). In such communities, members can speak without any restrictions, including the discussion of suicide behaviors and suicidal ideas. The largest of LDCs includes more than 600,000 members.

In view of China’s unique social and cultural background, this study aimed to explore how members use online depression communities and reveal the differences between LDCs and MDCs, so as to better support community members. This study evaluated the LDC and MDC in terms of (1) the themes of postings, (2) the type of members according to emotion expression and social support, and (3) the participation patterns of different groups of members.

## 2. Methods

### 2.1. Data Collection

In this study, the LDC under consideration comprises a comment thread appearing on Sina Weibo (Weibo, Beijing, China) [42], which is one of the most popular social media platforms in China, similar to Twitter (Twitter, San Francisco, U.S.A). In 2012, a Sina Weibo user named “Zoufan” committed suicide due to depression and posted a farewell message. For the past 8 years, there have been over 1 million comments under the farewell posting, and this number continues to grow. We chose this community as a data source because it is the largest and most representative LDC we know so far. Since the total number of comments under the farewell posting was too high, and premature data could not be obtained, we selected the comments from the previous year for our analysis. All comments from 1 January 2019 to 31 December 2019 (587,730 comments by 92,338 users) were crawled from the Zoufan community on Sina Weibo with the use of a spider that was programmed by Python (Python3.6.2, The Netherlands).

An MDC for comparative analysis had to be selected. We referred to some literature and surveyed some communities, including the depression community on Baidu Tieba (Baidu, Beijing, China) [15], DingXiangYuan [16], Sunshine [43], and Zhihu. Among them, the DingXiangYuan and Sunshine are online community platforms operated by medical and psychological service institutions and only for patients who have been diagnosed with depression. The number of posts and participants in online depression communities on DingXiangYuan and Sunshine is small. The Zhihu community platform belongs to the Q & A community, and the mode of communication is quite different from that of the Zoufan community. Baidu Tieba is the largest Chinese community in the world. The online depression community on Baidu Tieba and the Zoufan community on Sina Weibo have a wide range of users, similar modes of communication, fewer restrictions on user registration, and the same data scale. Therefore, we chose an online depression community on Baidu Tieba as the comparative data source. We obtained all posts (249,086 comments by 34,490 users) during the same time period (from 1 January 2019 to 31 December 2019).

An ethical review was not required because only publicly available data were used in this study.

### 2.2. Themes

In order to understand what users talk about in the LDC and MDC, and whether there are differences in information content between both communities, a thematic analysis of the postings was conducted. First, we preprocessed the whole text, including word segmentation and the removal of stop words. Keywords were extracted based on the term frequency–inverse document frequency (TF–IDF) [44]. Subsequently, we grouped and coded keywords and identified promising core themes. The coding method was equivalent to that used in qualitative research [45]. To better visualize the keywords, we drew keyword clouds.

### 2.3. Taxonomy of Emotion Expression and Social Support

In order to identify the type of emotion expression and social support, postings were classified. We divided emotions into three categories: positive (POS), neutral (NEU), and negative (NEG). Moreover, social support mainly includes two aspects: emotional support and informational support. A posting may express both emotional and informational support; thus, these two parts can be considered independently from each other. Therefore, we divided emotional support into seeking emotional support (SES), providing emotional support (PES), and if it was neither, we defined it as companionship (COM). Informational support was also divided into seeking informational support (SIS), providing informational support (PIS), and companionship (COM).

Given the impossibility to manually classify hundreds of thousands of texts, it was necessary to build text classifiers to identify the emotion and social support in postings. Classification was modeled as a supervised learning process where a training dataset was required. Due to the differences in language expression used in the two different community environments, and the different number of postings of each type in the annotated dataset of the two communities, the labeling process and the classifier building in the two communities were independent. Three research team members randomly selected 10,000 postings in the LDC and 10,000 postings in the MDC and independently labeled them. The majority rule was adopted to determine the assigned label when there was disagreement. We trained a text classifier using BERT (Bidirectional Encoder Representations from Transformers), a new language representation model developed by Google in 2018 that obtains new state-of-the-art results on 11 natural language processing tasks [46]. The Accuracy and F1 score were used to measure classifier performance. Table 1 shows the results of the labeling process, and Table 2 shows the performance of the classifier.

### 2.4. Member Profiling

After identifying the types of emotion and social support in each posting, we aimed to build profiles for all members of the communities by aggregating their postings by type. We considered that members may seek/provide emotional and informational support separately; thus, if emotional support and informational support were integrated, some characteristics of members may be hidden. Therefore, we represented each member’s emotion, emotional support, and informational support with three 1 × 3 vectors. Each element in the vector was the proportion of the member’ postings in the corresponding type. For example, if a member published 10 postings, namely 2 positive, 3 neutral, and 5 negative postings, then he/she will have the vector <0.2, 0.3, 0.5> for emotion. The same method was adopted for emotional and informational support.

After building the profiles for each member of the communities, we applied the classic *k*-means clustering algorithm to cluster members. The optimal number of clusters from the *k*-means clustering results was estimated using the R(R3.6.3) package NbClust (NbClust3.0) [47].

### 2.5. Participation Pattern

We investigated how members in different groups engaged in the communities. The level of engagement was measured by four metrics: productivity (i.e., members’ total number of postings), loyalty (i.e., number of days between members’ first and last posting), social activity (i.e., total number of replies to others), and attention received (i.e., total number of replies received). We compared the distribution of the four metrics between all clusters. To better clarify the differences between the clusters, the two-sample Kolmogorov–Smirnov test (K–S test) was used to compute the statistical gaps between every two clusters, with *p*-values <0.01 indicating statistically significant differences between clusters’ distributions of engagement metrics.

## 3. Results

### 3.1. Themes

**Figure 1:**
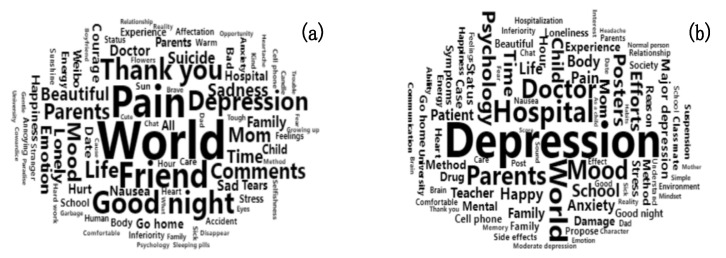
Keyword clouds in the lacking-management depression community (LDC) (**a**) and management depression community (MDC) (**b**).

Figure 1 displays the keyword clouds with the top 50 keywords by weight in the two communities. The weight calculated by TF–IDF is intended to reflect the significance of the word to a document in a corpus. The keywords for each group were coded to determine the representative themes. The themes in the LDC were usually about the expression of emotions and feelings, mainly negative emotions (e.g., pain, sadness, depression) and daily communication (e.g., friend, good night, thank you). The themes in the MDC were mainly the treatment of depression (e.g., depression, hospital, doctor) and personal life (e.g., parents, child, school).

### 3.2. Emotion Expression and Social Support in Postings

**Figure 2:**
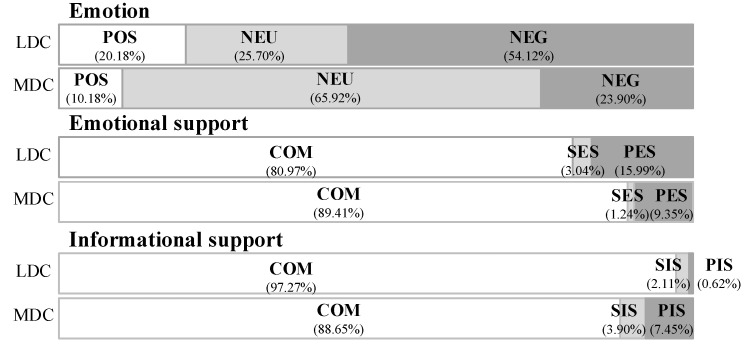
Proportion of postings of each type in the management depression community (MDC) and lacking-management depression community (LDC).

Figure 2 shows the proportion of postings of each type in the two communities after classification. Regarding emotion, it can be seen that more than half of the postings in the LDC expressed negative emotion, while in the MDC, most postings expressed neutral emotion. In terms of social support, postings regarding emotional support in the LDC were more than those in the MDC, and postings regarding informational support in the MDC were more than those in the LDC.

### 3.3. Member Profiling

**Figure 3:**
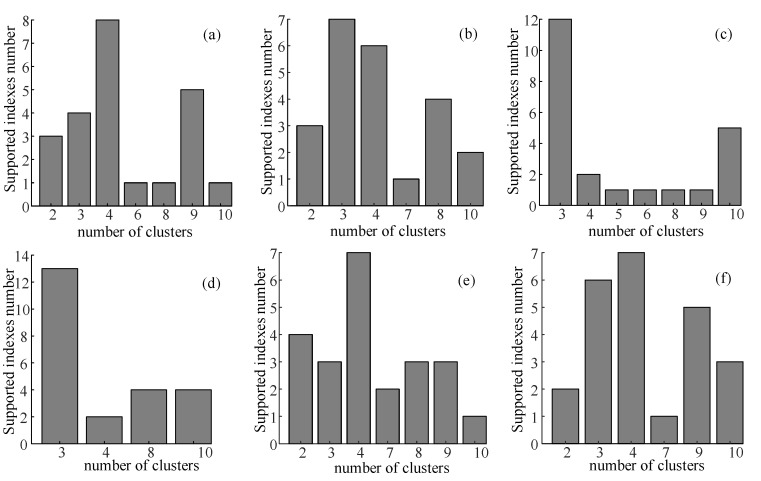
Optimal numbers of members’ clusters in the management depression community (MDC) regarding (**a**) emotion, (**b**) emotional support, and (**c**) informational support; optimal numbers of members’ clusters in the lacking-management depression community (LDC) regarding (**d**) emotion, (**e**) emotional support, and (**f**) informational support.

After identifying posting types, we built member profiles by type. To determine the emotional characteristics of members and the different roles they played in emotional and informational support, members’ profiles in the two communities were clustered. Figure 3 presents the optimal number of clusters from the *k*-means clustering results. The clustering results and proportions of groups in each type are given in Table 3. There were more members with extreme negative emotions in the LDC than that in the MDC. The number of members in the companionship group was the largest in both emotional support and informational support. There were more emotional support providers and seekers in the LDC, while there were more informational support providers and seekers in the MDC. In general, there were more support providers than support seekers in the two communities. However, in the LDC, there were less informational support providers than informational support seekers. These results reveal the differences in terms of member types between the two communities.

### 3.4. Participation Patterns

After determining the types of members, in order to better understand their use of online communities, we investigated the participation patterns of different types of members using the defined engagement metrics.

#### 3.4.1. Emotion

**Figure 4:**
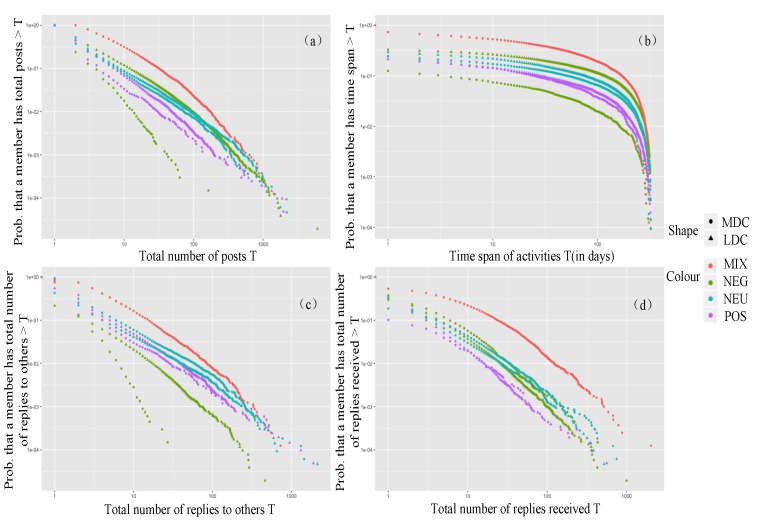
Complementary cumulative distributions of engagement metrics for the different clusters of emotion expression: (**a**) productivity, (**b**) loyalty, (**c**) social activity, and (**d**) attention received.

The community engagement of each group in terms of emotion is shown in Figure 4. In the LDC, the productivity and loyalty of the negative emotional group were the highest of all the groups and they did not actively communicate but received high attention. The positive emotional group was not active in the community. In the MDC, the mixed group was the most active of all the groups; the productivity, loyalty, and social activity of the negative emotional group were significantly lower than those of other groups. It can be seen that members with negative emotion were more dependent on the LDC.

#### 3.4.2. Emotional Support

**Figure 5:**
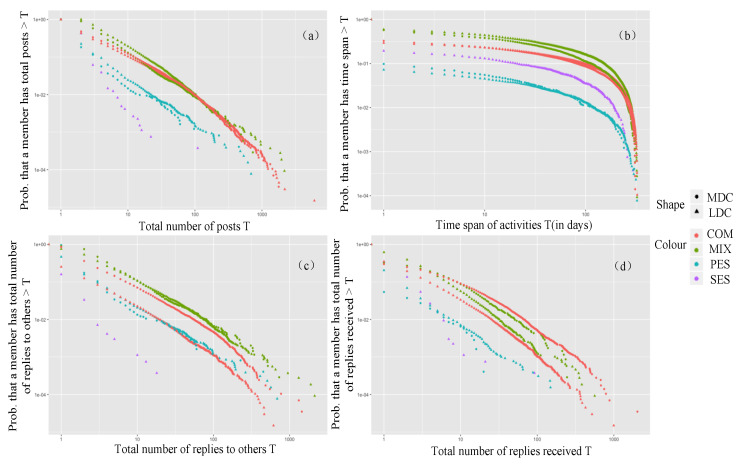
Complementary cumulative distributions of engagement metrics for the different clusters of emotional support: (**a**) productivity, (**b**) loyalty, (**c**) social activity, and (**d**) attention received.

As shown in Figure 5, the emotional support seekers and providers in both communities were not active. Among them, the productivity, social activity, and attention received of emotional support providers in the LDC were higher than those of the seekers. Although emotional seekers spent much time in the LDC, they did not receive attention. This may be due to their low level of productivity and social engagement in the LDC. In the MDC, there was no obvious division of emotional support seekers.

#### 3.4.3. Informational Support

**Figure 6:**
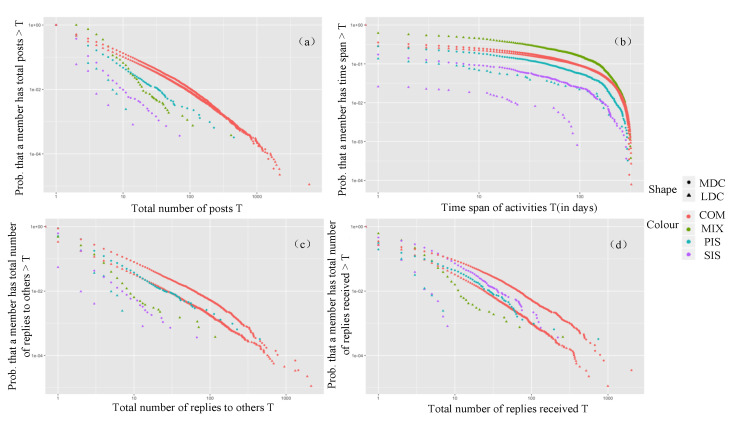
Complementary cumulative distributions of engagement metrics for the different clusters of informational support: (**a**) productivity, (**b**) loyalty, (**c**) social activity, and (**d**) attention received.

As shown in Figure 6, the information seekers and providers in the MDC scored significantly higher than those in the LDC in the four engagement metrics; the productivity, loyalty, and social activity of information providers in the MDC were significantly higher than those of seekers, while the attention received by seekers was higher. This suggests that the MDC established a good platform to provide informational support for members. However, the productivity and social activity of information providers in the LDC were low, and the attention received by information seekers was low, suggesting that information sharing in the LDC was poor.

## 4. Discussion

This study revealed the potential risks of LDCs by discussing the themes of postings, types of members, and participation patterns of member types in the LDC and MDC. This knowledge is important in order to provide better support for members of LDCs.

Firstly, we found through content analysis that the most common themes in the LDC were negative emotion and daily communication, while in the MDC, the most common themes were treatment of depression and personal life. Treatment information about depression in the MDC was found to help members deal with the disease actively [37], while serious negative emotions and chats about suicide in the LDC were found to lead members to extreme behaviors. Previous studies have shown that discussions about suicide on online communities increase users’ future suicide risk [48]. Therefore, a system could be put in place to monitor community content by setting up a text warning system, and when possibly damaging statements appear, such as those on suicide, a warning would appear. In addition, we should actively and positively guide the expression of emotion of members in LDCs and increase the amount of information regarding treatment of depression to make it available to more members.

Secondly, we divided members based on the results of the text classification. We found that the LDC had the largest number of members in the negative emotional group. Previous research found that frequent expression of negative emotions on social media is associated with higher levels of depression symptoms [26,27,28]. Our results may help to identify patients with severe depression so that they can be prioritized. In addition, we also proposed detailed engagement metrics to describe the participation patterns of members. We found that the negative emotional group posted the most and spent much time involved in the LDC. This pattern suggests that people with negative emotion may be more dependent on LDCs, and help from the LDCs may be more acceptable to negative emotion groups. According to emotional contagion theory [49], a person’s emotion spreads to other people during social encounters. Depressive symptoms and negative emotions too have been found to be contagious both via social media and personal contact with strangers or acquaintances [50,51,52]. The reason for the large number of group members with negative emotions in LDC may be the aggregation of individuals with depression. However, it may also be due to the spread of negative emotions. Conversely, support for negative emotion groups can reduce the occurrence of emotional contagion and may also indirectly help others, which suggests the low cost and high return of the intervention. In addition, attention should also be paid to the emotional changes among users who interact with negative emotion groups over a long period of time.

Thirdly, previous studies have suggested that communities provide more emotional support than informational support [9,33]. However, these studies did not consider the impact of community diversity. Our study found that although sharing stories from personal daily life and activities not directly related to depression were key for keeping members together, members also exchanged different types of social support in both communities. In terms of emotional support, the results showed that in the LDC, the number of emotional providers was higher than that of seekers, but providers only stayed in the community for a short time. Some of these providers may be volunteers. Thus, we suggest that communities should take measures to attract and sustain long-term participation. In terms of informational support, the results showed that in the MDC, the number of providers was higher than that of seekers, providers were active, and seekers also received high attention. On the other hand, in the LDC, the number of providers was very small, and information seekers could not easily receive information. The reason for this difference could be that some of these providers are part of a support group, such as the mental health service staff, who will promptly respond to the information seekers. There was a lack of support groups in the LDC, which leads to the inability of community members to receive professional medical help. More information sharing will promote the healthy development of communities and provide effective support to their members. Therefore, we suggest the following measures. First, it is necessary to introduce support groups to LDCs. Secondly, considering the scarcity of mental health service staff in China [41], by establishing a connection between MDCs and LDCs, LDC members could enjoy the resources of the MDC through resource sharing. Finally, as it is more acceptable for patients to find support from people with the same disorder [53,54], experienced patients should be solicited to work with support groups and contribute valuable information about depression. In addition, the identification of seekers of different types of social support can also help community managers to support seekers more specifically.

Finally, there are some limitations to this study. First, we revealed the risks associated with LDCs, but whether LDCs have a negative impact on their members has not been confirmed, as our study was based on cross-sectional data. Therefore, a follow-up study using longitudinal data could establish the impact of community participation on members. For example, whether the members’ emotion improves or deteriorates after participating in the LDC or how the interaction with negative emotion groups affects members’ future emotional expression could be examined. We can also ascertain which theme information can result in an improvement or deterioration in members’ emotions easily, and which members are more likely to be influenced by the emotions of the members with whom they interact. The impact of receiving emotional support and/or informational support on members’ future emotional expression and participation can also be studied to provide more suggestions for community management and help community members better. Second, in this study, two community data sets were used for the comparative analysis, and there is a possibility of selection bias. More samples should be included in the follow-up study, and other community types, such as clinical depression communities, should be added. The differences in members’ expression and participation and the impact on members in different communities should also be analyzed and compared.

## 5. Conclusions

Our study provides new insights to explore how members use online depression communities. The potential risks of LDCs were revealed through a detailed comparative study of the LDC and MDC. The findings suggest that there was a large number of individuals with extreme negative emotions in the LDC. This indicates higher levels of depression symptoms in these members at a broad level. However, there was a lack of effective information sharing about the treatment of depression in this community. This may be due to the lack of information providers, such as support groups, in the LDC. Moreover, this community provided limited emotional support for its members. The results of our study confirm the need for community management. Our findings may also be used to develop more efficient measures to support members of online depression communities.

## Figures and Tables

**Table 1:** Proportion of postings of each type in the annotated dataset of the management depression community (MDC) and lacking-management depression community (LDC).

Community	Emotion		Emotional Support		Informational Support	
	Type	Proportion	Type	Proportion	Type	Proportion
LDC	POS	20.03%	COM	80.28%	COM	96.24%
	NEU	50.48%	SES	3.11%	SIS	2.72%
	NEG	29.59%	PES	16.61%	PIS	1.04%
MDC	POS	11.76%	COM	87.31	COM	89.01%
	NEU	62.14%	SES	1.92%	SIS	4.28%
	NEG	26.10	PES	10.77%	PIS	6.71%

POS-positive, NEU-neutral, NEG-negative, SES-seeking emotional support, PES-providing emotional support, COM-companionship, SIS-seeking informational support, PIS-providing informational support.

**Table 2:** Performance of classifier of emotion and social support in the management depression community (MDC) and lacking-management depression community (LDC).

Community	Emotion (%)		Emotional Support (%)		Informational Support (%)	
	Accuracy	F1 score	Accuracy	F1 score	Accuracy	F1 score
LDC	94.48	96.52	93.38	84.79	97.58	74.56
MDC	86.67	81.83	95.10	81.36	95.57	81.00

**Table 3:** Clustering results and proportion of groups in each type.

**Emotion**			
	**Centroids (NEU, POS, NEG)**	**Type**	**Proportion (Number)**
LDC	<0.049, 0.034, 0.917>	Negative emotional group	55.67% (51,487)
	<0.768, 0.086, 0.146>	Neutral emotional group	23.11% (21,342)
	<0.037, 0.853, 0.109>	Positive emotional group	22.21% (20,509)
MDC	<0.969, 0.017, 0.013>	Neutral emotional group	48.90% (16,377)
	<0.532, 0.066, 0.402>	Mixed emotional group	23.99% (8033)
	<0.016, 0.005, 0.979>	Negative emotional group	20.01% (6701)
	<0.136, 0.8241, 0.040>	Positive emotional group	10.09% (3378)
**Emotional support**			
	**Centroids (COM, SES, PES)**	**Type**	**Proportion (number)**
LDC	<0.979, 0.0101, 0.011>	Companionship group	72.46% (66,905)
	<0.015, 0.001, 0.984>	Emotional support providers	14.01% (12,940)
	<0.558, 0.023, 0.420>	Mixed group	11.72% (10,822)
	<0.174, 0.800, 0.027>	Emotional support seekers	2.89% (2671)
MDC	<0.985, 0.003, 0.012>	Companionship group	85.30% (28,565)
	<0.516, 0.186, 0.298>	Mixed group	10.33% (3461)
	<0.024, 0.001, 0.975>	Emotional support providers	7.35% (2463)
**Informational support**			
	**Centroids (COM, SIS, PIS)**	**Type**	**Proportion (number)**
LDC	<0.996, 0.003, 0.001>	Companionship group	96.42% (89,034)
	<0.668, 0.309, 0.022>	Mixed group	2.88% (2659)
	<0.011, 0.989, 0.0001>	Informational support seekers	1.34% (1234)
	<0.208, 0.011, 0.781>	Informational support providers	0.45% (411)
MDC	<0.962, 0.016, 0.022>	Companionship group	85.57% (28,656)
	<0.175, 0.034, 0.791>	Informational support providers	9.21% (3085)
	<0.141, 0.852, 0.007>	Informational support seekers	8.21% (2748)
